# Deep Learning-Based Object Detection for Unmanned Aerial Systems (UASs)-Based Inspections of Construction Stormwater Practices

**DOI:** 10.3390/s21082834

**Published:** 2021-04-17

**Authors:** Billur Kazaz, Subhadipto Poddar, Saeed Arabi, Michael A. Perez, Anuj Sharma, J. Blake Whitman

**Affiliations:** 1 Department of Civil Engineering, Auburn University, Auburn, AL 36849, USA; mike.perez@auburn.edu; 2 Department of Civil, Construction and Environmental Engineering, Iowa State University, 813 Bissell Road, Ames, IA 50011, USA; spoddar@iastate.edu (S.P.); arabi@iastate.edu (S.A.); anujs@iastate.edu (A.S.); 3 Pitt-Des Moines, Inc., Elk Grove, CA 95624, USA; 4 School of Concrete and Construction Management, Middle Tennessee State University, Murfreesboro, TN 37132, USA; blake.whitman@mtsu.edu

**Keywords:** construction stormwater management, inspections, unmanned aerial systems, photogrammetry, deep learning-based object detection

## Abstract

Construction activities typically create large amounts of ground disturbance, which can lead to increased rates of soil erosion. Construction stormwater practices are used on active jobsites to protect downstream waterbodies from offsite sediment transport. Federal and state regulations require routine pollution prevention inspections to ensure that temporary stormwater practices are in place and performing as intended. This study addresses the existing challenges and limitations in the construction stormwater inspections and presents a unique approach for performing unmanned aerial system (UAS)-based inspections. Deep learning-based object detection principles were applied to identify and locate practices installed on active construction sites. The system integrates a post-processing stage by clustering results. The developed framework consists of data preparation with aerial inspections, model training, validation of the model, and testing for accuracy. The developed model was created from 800 aerial images and was used to detect four different types of construction stormwater practices at 100% accuracy on the Mean Average Precision (MAP) with minimal false positive detections. Results indicate that object detection could be implemented on UAS-acquired imagery as a novel approach to construction stormwater inspections and provide accurate results for site plan comparisons by rapidly detecting the quantity and location of field-installed stormwater practices.

## 1. Introduction

Temporary erosion and sediment control (E&SC) practices on construction sites provide protection for the downstream environment by minimizing the impact of sediment-laden stormwater runoff associated with land-disturbing activities. Active construction sites are susceptible to increased erosivity due to grading and land-disturbing activities that often expose multiple acres of land. These disturbed areas are a potential risk to release large amounts of sediment into existing water bodies [1]. According to the U.S. Environmental Protection Agency (USEPA), construction sites cause an annual loss of up to 247 tons/ha (100 tons/ac) in the U.S. [2]. Anthropogenic-associated sediment discharge creates environmental and ecological risks and results in the destruction of fish habitat, degradation of water quality, and reduces the capacity of streams, harbors, and rivers [3,4]. Through the Clean Water Act, the National Pollutant Discharge Elimination System (NPDES) permit regulates point and nonpoint sources of pollution and obligates operators of construction activities to file for a Construction General Permit (CGP) [5]. NPDES regulations require routine inspections once every seven calendar days or every 14 days within 24 h after the occurrence of a storm event to ensure construction stormwater practices are in place and performing as intended [4]. NPDES permit reporting calls for inspections to be documented in a formal report and stored for a period of three years following the conclusion of the construction project.

Common construction stormwater practice deficiencies include improper placement (i.e., installing practices in a location where the practices would not be effective), ineffective installation technique, inadequate fastenings, and incorrect post spacing. Vulnerabilities observed during inspection may trigger the need for design modifications, due to changing site conditions with ongoing construction activities. Construction stormwater inspectors detect these vulnerable areas and deficiencies to determine the need for corrective actions. Traditionally, inspections have been performed on-foot, requiring inspectors to traverse the entire jobsite to inspect every construction stormwater practice installed on-site. Often, on-foot inspections and subsequent documentation are inadequate on linear and highway construction projects due to their overall scale and challenging terrain. In addition, the frequency of inspections can be burdensome on the limited inspection personnel that are often tasked with several other duties on a jobsite. Identifying the location and the number of the E&SC practices can be challenging for stormwater inspectors as construction plan sets usually use tables or simple plan views to describe where practices should be installed. Often, construction activities require the use of additional E&SC practices or modification of the original SWPPP. Any changes or deviation to the SWPPP must be carefully recorded by a qualified inspector. Proper inspection and record-keeping can be laborious in large or linear construction sites. Current record-keeping procedures consist of paper or web-based inspection forms, and ground-based images. Moreover, locating the same practice on the site to inspect if corrective actions have taken place can be challenging with changing site conditions and lack of detail in typical inspection reports and site plans. Audits by regulatory agencies often find deficiencies in inspection and reporting, resulting in hefty fines and stop-work orders. A need exists to provide effective installation and recording procedures to assist inspectors in conducting comprehensive construction stormwater inspections. An opportunity to leverage aerial inspection tools has the potential to provide an effective approach for improving construction stormwater inspection procedures.

Unmanned Aerial Systems (UASs) can be defined as a system that includes unmanned aerial vehicles (UAVs) and related sensors and equipment that connects and controls the aircraft [6]. Various sensors, such as consumer-grade cameras, thermal cameras, hyperspectral cameras, and laser scanners, are compatible with UAVs [7]. UASs have quickly flooded the inspection market as a remote sensing tool, capturing spatial data at a high temporal resolution. Through rapidly developing technology, UASs are playing an influential and vital role in meeting the demands of various professional fields including military, agriculture, disaster management, and construction [8]. The construction industry in particular is a beneficiary of this technology with applications in visual observation and documentation on construction projects. These systems have the ability to capture a vast number of high-resolution images and process data into three-dimensional (3D) models, which serve the construction industry in applications of surveying, monitoring, and inspecting inaccessible locations on construction sites [9]. For example, researchers have investigated the use of UASs for implementing structural inspections [10], conducting site inspections for erosion and sediment control practices [11], calculating earthwork volumes [12], developing thermography surveys [13], and determining risk factors with proximity detection [14].

Another emerging technology is deep learning-based object detection, a computer vision science that predicts the location of objects by using classification and localization tasks [15]. It has been used to automate various applications such as image retrieval, security, vehicle detection, person detection, surveillance, and machine inspection, etc. [15]. Researchers utilize deep learning-based approaches to solve a variety of problems in civil engineering that includes structural damage detection [16], crack detection [17], construction equipment detection [18], and automated vehicle recognition [19].

This study implements the use of UAS inspection technology coupled with deep learning-based object detection to provide an innovative approach to construction stormwater inspection tasks. The developed system focuses on solution creation, which consists of data preparation, model training, validation, tests, and post-processing steps. To evaluate the developed system, a total of 18 aerial inspections were conducted on a 19 km (12 mi) roadway widening project on U.S. Highway 30 in Tama County, Iowa. The project included large areas of ground disturbance with thousands of temporary construction stormwater practices installed throughout the site. Object detection was successfully performed on a total of three types of E&SC practices: rock check dams, wattle ditch checks, and silt fence used in both ditch checks and sediment barrier applications. Over the course of the 2019 construction season, approximately 6000 georeferenced photographs were captured with a Zenmuse X5S^TM^ camera mounted on a DJI Inspire 2^TM^ quadcopter. These images were used in the data set preparation process, which used a framework of photogrammetry, GIS, and image labeling applications. Single-shot Multibox detectors (SSD) were used as a detection model. SSD is a mechanism where multiple objects are detected in an image using a single look at the image by the model, unlike those of the RCNN family where once it is used for detection and other time for proposal. The idea of the SSD is to predict the score for different categories of objects and box offsets for a specific number of default bounding boxes using small convolution filters applied to feature maps [20]. It also uses the concept of non-maximum suppression in order to predict the final location and number of objects in an image.

Image-based object detection has become prominent in the construction industry. Arabi et al. used a modified version of the SSD-MobileNet object detector and detected construction equipment for improving safety on sites [18]. Zhang et al. integrated deep-learning principles with three-dimensional (3D) laser imaging technology to identify cracks on asphalt surfaces [21]. Chakraborty et al. implemented You Only Look Once (YOLO) and Deep Convolutional Neural Network (DCNN) on camera imagery and detected traffic congestion [22]. Cha et al. aimed to automate structural inspection procedures of civil infrastructures by presenting an image-based structural damage detection framework that utilizes Faster Region-based Convolutional Neural Network (Faster R-CNN) [23]. Faster R-CNN was also adopted by Fang et al. for detecting non-hard-hat use and safety harness detection on construction sites [24,25]. All of these research studies implemented image-based object detection principles with different detection models to advance and automate current applications in the construction industry. The presented study carries a similar purpose with these studies and introduces object detection principles to a novel application, construction stormwater inspections. Although the presented work has a similar motivation to that of previous studies on bringing automation into the construction industry, it utilizes a unique detector that has not been used in the prior studies. Visual Geometry Group-16 Single Shot Detector (VGG 16- SSD) was used in this presented work to detect temporary E&SC practices. Wu et al. follow a similar detection framework to the presented study by using VGG 16-SSD for automatic detection of hardhats worn by the construction personnel. The results indicated that the detection can achieve 83.89% Mean Average Precision (MAP) for the input image size of 512x512 pixels [26]. However, this study used Internet-derived images for model training, which is a different data set source than the presented study.

The detection of E&SC practices on construction sites enables inspectors and designers to easily and accurately compare the stormwater pollution prevention plan sets with site applications. Our literature review revealed that prior studies have not utilized object-based detection for construction stormwater inspections. The proposed methodology brings an innovative and novel approach to stormwater inspections on construction sites by using deep learning-based object detection principles. The presented study demonstrates how UAS-acquired imagery can be easily used as a dataset for object detection and provide accurate results for construction stormwater inspections. This research provides inspectors with implementable guidance on the use of UAS and software technology to identify E&SC deficiencies throughout a site. This approach advances aerial inspections by increasing the efficiency and quality of inspection results. Moreover, this work contributes to inspection procedures by decreasing inspection times and providing comprehensive documentation.

## 2. Methodology

**Figure 1:**
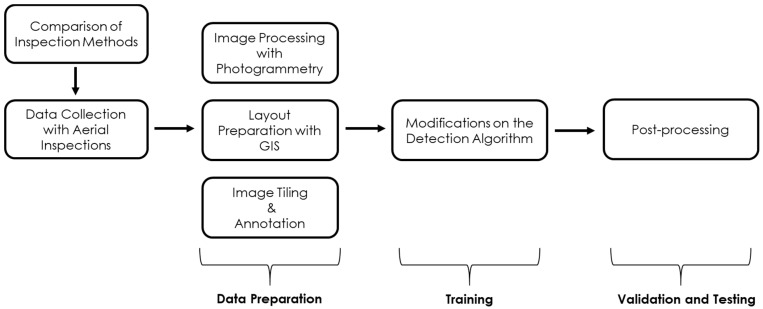
Flow chart of the methodology.

This study compared aerial inspection methods to on-foot traditional inspections for identifying the limitations and challenges. Aerial images were chosen as a data source for object detection as a result of this comparison. Figure 1 illustrates the order of each step in the methodology. Data collection was followed by a data preparation procedure, which included the use of photogrammetry, GIS, and annotation tools. Model training required modifications to an existing detection algorithm, VGG 16-SSD [27]. The final step was validation and testing which included a post-processing step that merges the detected dataset and validates the accuracy of the model.

### 2.1. Aerial Inspections

Aerial inspections required a UAS unit that had sufficient technical capabilities for weather conditions and the ability to use different cameras and sensors. To translate this research into practical application, it was important to use readily available hardware and software platforms. The DJI Inspire 2^TM^ was preferred as a UAS system due to its relatively inexpensive cost, 27-min battery life, and a lock-and-go gimbal feature for using different types of cameras [28]. In addition, DJI Inspire 2^TM^ provided a comfortable flight experience with its capability for flying in a range of temperatures (−4 °F to 104 °F) and its well-developed global positioning system (GPS), which assists in-flight management and tracking [28]. Camera quality was another important consideration when developing highly effective products with the UAS system. The Zenmuse X5S **^TM^** camera was selected for this study, capturing 16 MP high-resolution images with its 360-degree rotatable gimbal and auto-calibration features [29]. The selected UAS system included a UAV, eight propellers (including four spare propellers), twelve batteries, two charging hubs, battery heat insulators, a remote controller, and an iPad Pro 10^TM^ tablet. The second step of the aerial inspection methodology development was to determine the appropriate automated flight applications that were compatible with the UAS system and photogrammetry software. Georeferenced images were captured using automated flight applications and were exported into photogrammetry software. A mobile application, Pix4D^TM^ Capture, was used to configure different flight missions for different purposes, such as creating 2D maps, 3D models, and a single 3D model. Polygon- and grid-shaped flight patterns were used for creating 2D maps, while double grid- and circular-shaped flight patterns were used for 3D models. In this study, double grid flight missions were used to create more precise models due to the number of images that overlap. However, for flight efficiency, single grid flight missions were used during flights that covered large distances over 4 ha (10 ac). Ground Control Points (GCP) were used to support the correction of uncertainties in image geolocation. Eight GCPs were prepared for this study by creating 0.6 m × 0.6 m (2 m × 2 ft) plywood markers and painting with black and white markings. The GCP markers were diagonally spread across the site in pairs and a Trimble R8 Real-Time Kinematic (RTK) surveying unit was used to obtain northing, easting, and elevation information of the GCP markers.

All flights were conducted by the Federal Aviation Administration (FAA) certified remote pilots who received the required permissions to conduct flights in the area. During the course of construction activities from March 2019 to October 2019, 18 flights were conducted on the site at different locations. Each automated flight typically reached 91 m (200 ft) flight altitude and captured over 700 images that were used to develop orthomosaic models of the site. These images were used as datasets in the development of deep learning-based object detection to detect temporary E&SC practices such as rock check dams, silt fences, and wattles installed on site. In addition to automated, pre-programmed flights, manual flights were also conducted to focus on failures or deficiencies on-site. Aerial inspections were compared to traditional on-foot inspections to better understand the limitations in the traditional approach. UAS-based images and ground-based images of practices installed on-site were compared to identify the differences in the information that can be interpreted about deficiencies and the location of the practices. This comparison showed the differences in both perspectives and provided an understanding of how aerial inspections provide comprehensive record keeping. Based on this comparison, aerial imagery was chosen as a valid data source for object detection.

### 2.2. Data Preparation

Photogrammetry enables the development of accurate and high-resolution 3D object models by using multi-image stitching techniques [30]. Photogrammetry applications precisely rectify and overlap georeferenced aerial images into mosaics [31,32,33]. Typical products of photogrammetry include Digital Surface Models (DSMs), contours, vector data, and 3D models. To generate accurate products, proper data collection is required, which calls for careful planning and consideration of flight missions along with calibration and image triangulation [32]. This study used Pix4D^TM^ Capture to plan automated flight missions and enable the UAS to take georeferenced images by following a predetermined path [34]. Post-data collection processing was conducted on a desktop computer using Pix4D^TM^ Mapper, a software package that uses image stitching to produce 3D models by using georeferenced aerial images. This software uses Automatic Aerial Triangulation (AAT) and Bundle Block Adjustment (BBA) to accurately match georeferenced images [35]. AAT increases the accuracy of image stitching by using aerial triangulation as a serial execution process, while BBA optimizes the images for 3D model reconstruction [36,37].

**Figure 2:**
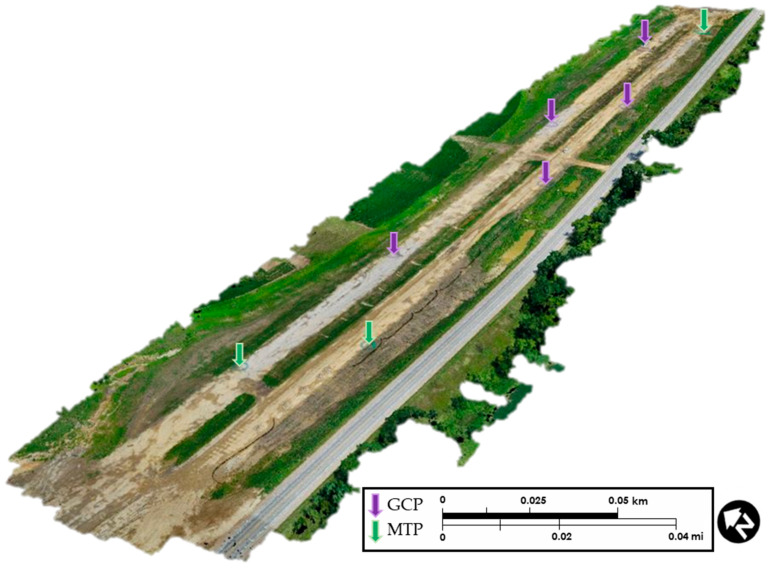
Re-optimized site model with GCPs and MTPs.

Georeferenced aerial images collected during flights were stitched in three processing steps (initial processing, point cloud, and 3D mesh production and DSM, and orthomosaic production) and a 3D map of the survey area was created. Sixteen different models of the site were produced by using this photogrammetry processing tool for various locations of the construction site. Seven of the processed models served for deep learning-based object detection applications. The Pix4D^TM^ Mapper software required initial input data prior to initiating data processing, including information related to the camera model, image upload coordinate system, and map type. The coordinate system used for all models was kept consistent at Iowa North NAD83, the local projection system. The images were uploaded in .jpg format and EXIF data, which specifies formats for images. Initial processing created matches between hundreds of images and used AAT and BBA methods for optimizing the images in a way that increased the accuracy of the orthomosaic. At the completion of the initial processing step, quality reports were provided to show the accuracy of the image stitching. These quality reports presented the need for increasing image location accuracy since each model had approximately 1.8 m (6 ft) of error in elevation, northing, or easting. This error was calculated in the quality reports by using mean, standard deviation, and root mean square methods in each direction (X, Y, Z). After completion of initial processing in Pix4D^TM^ Mapper, the sequence was interrupted for incorporating GCPs into the model. Additional manual tie points (MTPs) were incorporated into the model. Each MTP had at least eight images that included the selected GCP. Re-optimization was conducted to match the model with GCPs by readjusting camera parameters. An example result of the re-optimized model is provided in Figure 2. Arrows indicate the location of GCPs (purple) and MTPs (green).

The second processing step created a densified point cloud view of the area of intent by using additional tie points, which increased the accuracy of the model. The point cloud represents data points related to the surface view, which are created by using the image stitching technique. The third processing step created a DSM and an orthomosaic view of the area of intent in tagged image file format (.tiff), which can be imported into the geographic information system (GIS) or computer-aided design (CAD) software for further analysis. These outputs were imported into ArcMap^TM^ 10.5.1 by using the same coordinate and projection system for conducting analysis on the surfaces. The imported orthomosaic views were displayed as high-resolution scaled images and provided a dataset for site plans and deep learning-based object detection applications.

#### 2.2.1. Layout Preparation

GIS applications were used to create scaled layouts of orthomosaic views that were created for various locations on the study site, capturing various types of temporary E&SC practices in a single view. Orthomosaic files were displayed as color-mapped raster layers in ArcMap^TM^ that showed detailed practices on the site models. First, orthomosaics were displayed in the data view, which enabled editing and analyzing the data. Prior to layout preparation, scaling accuracy was verified using measurements of known landmarks on the orthomosaics to validate developed models.

**Figure 3:**
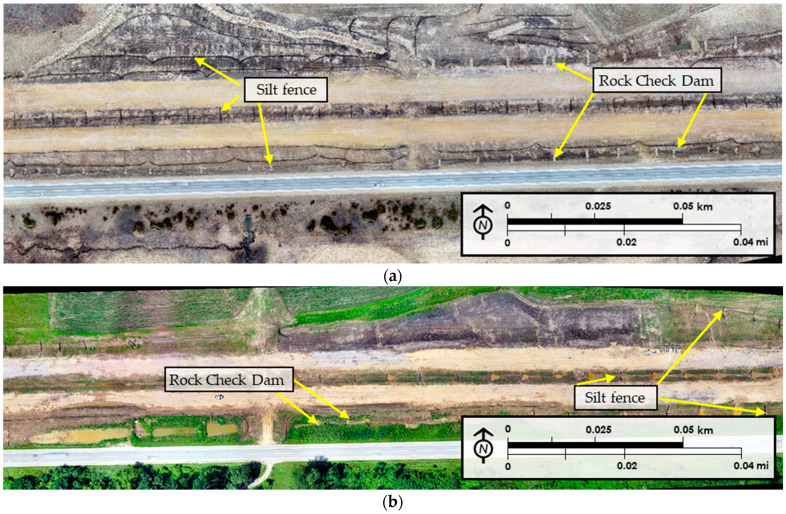
U.S. 30 highway construction site layout views: (**a**) 28 March 2019: (**b**) 18 July 2019.

Figure 3 illustrates examples of exported layouts based on the orthomosaic view. Figure 3a shows a layout that was prepared at the beginning of the construction season in March of 2019. The image easily depicts rock check dams and silt fence, which were used in both sediment barrier and ditch check applications during this stage in the construction process. Figure 3b shows a different location on the site during August of 2019. During this phase, established vegetation is visible, along with silt fence ditch checks and sediment basins. Later, during the construction season, silt fence sediment barriers and wattle ditch checks were observed in the same area. Therefore, another layout of the same location was used to train the object detection model together with this layout shown in Figure 3b. As a result of layout preparation, rock check dams, wattle ditch checks, and silt fence were determined as the temporary E&SC practices to be detected in this study due to their frequency in the application on the site. GIS data was prepared and exported using scaled and rectified views for use in deep learning-based object detection applications. To develop a robust detection model, a high number of practices are needed to increase the percentage of detection accuracy with an effective validation dataset. Validation dataset should be formed by using at least 20–25% of the entire dataset [18]. Hence, seven different layouts which included at least 200 (i.e., at least 20% of the original dataset) construction stormwater practices were prepared. Layouts were exported in .jpg format with 600 dpi resolutions.

#### 2.2.2. Image Tiling and Labeling

**Figure 4:**
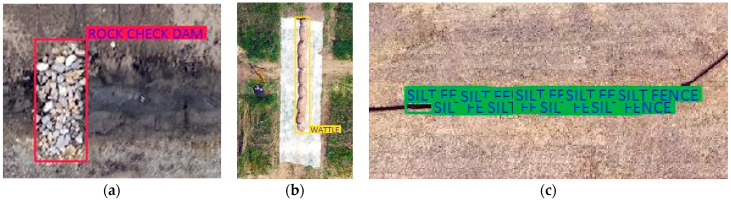
Annotation of temporary E&SC practices: (**a**) rock check dam; (**b**) wattle ditch check; (**c**) silt fence sediment barriers.

A Single Shot Multibox Detector (SSD) model was used in this study to detect construction stormwater practices visible on the UAV acquired imagery. Image tiling was required to prepare images for annotation, a necessary step in training the model. SSD images are based on 300 × 300 pixels size so tiling was done to ensure that the objects were properly visible for detection and identification by the algorithm. Python programming language was used for annotating images, with files formatted as .json file format. Visible target objects were annotated using rectangular boundaries and labeled according to their names. Annotation borders were created as close as possible to the observed stormwater practice to increase the accuracy of the training procedure. The .json files provided information on the annotated bounding boxes around the practices by displaying pixel values on the image. A total of 800 labeled .json files were found to be sufficient to feed the object detection model for it to become capable of detecting practices with accuracy. Rock check dams and wattle ditch checks were labeled using single rectangle-shaped boxes enveloping the entire object within. However, due to their linear shape, silt fence sediment barriers required multiple small rectangle boxes placed as close as possible to the practice. Annotated silt fence boxes were joined through the entire silt fence to represent a single object later in the post-processing step by using clustering. Figure 4 shows examples from the annotation procedure for different practice types: rock check dams, wattle check dams, and silt fence. After the annotation step, the data set was ready for model training.

#### 2.2.3. Model Development

Object-based image detection has been extensively studied over the past several years and has been applied in several fields of science and engineering [38,39,40]. Ideally, there are three categories of object-based image detection—You Only Look Once (YOLO) [41], Region-Based Convolutional Neural Network (RCNN) [42], and Single Shot Multibox Detector (SSD) [20]. SSD models have the advantage of binding both localization and detection tasks in a single wipe across the network, resulting in significantly faster detections, making it easily deployable on lower configuration hardware. The SSD is a pure convolutional neural network, which consists of three main processes as follows:

- Base convolutions derived from modified VGG-16, a convolutional network for classification and detection, where the weights of the traditional VGG-16 models were enhanced by transfer learning [27] that provided lower-level feature maps. Some of the notable changes to the pre-trained VGG-16 network to make it adaptable to the object detection by SSD are as follows:
  - The input size of the images was fixed to 300 by 300 pixels.
  - The third pooling layer of the VGG-16 network was converted from a floor function to a ceil function. This was done to compute the feature map of 75 × 75 into 38 × 38 instead of the default 37 × 37 which would have made calculations difficult at a later stage.
  - To prevent the halving of the feature maps from the previous layers, the fifth pooling layer used a 3 × 3 convolution kernel with a stride of 1, which was a 2 × 2 convolution kernel with a stride of 2 in the original study.
  - As classification was not the primary objective of the base convolution layer, the fully connected layer 8 (fc8) was removed and the fc6 and fc7 were replaced by the respective convolutional layers.
- Auxiliary convolutions were added on top of the base network that provided higher-level feature maps.
- Prediction convolutions that used localized predictions and prediction of the classes in these feature maps to detect the location and the type of the object(s) in the images were finally applied.

Using this concept, the model was trained using the SSD technique. A multi-box loss was developed as there were two types of predictions that were necessary for model training. This multi-box loss encompasses losses due to regression of the bounding boxes and the classification of classes into a single entity. The regression loss of the location loss consisted of the L1 norm loss, while the classification loss was the sum of the Cross-Entropy losses among the positive and hard negative losses [27]. Hard positives represent the images that have the object in them, and hard negatives represent images that do not contain the object in them. Mathematically, the averaged Smooth L1 norm loss between the encoded offsets of positively matched localization boxes and their ground truths were written as shown in Equation (1). To determine the classification loss, hard negatives and positives were used. The number of hard negatives (N_hn_) was taken to be three times that of the hard positives (N_p_). The most difficult hard negatives were identified by finding the Cross-Entropy Loss for each negatively matched prediction and then choosing those top N_hn_ losses. The confidence loss can be determined as shown in Equation (1).

(1) $$L_{loc}=\frac{1}{n_{positives}}(\sum_{positives}Smooth\;L_{1}\;Loss)$$

where

*L_loc_* = *localization loss*
*n_positives_* = *total number of positives*
*Smooth L*_1_*Loss* = *the smoothened L*_1_ *loss between the encoded offset of the positively matched localization box with their ground truth*

The final evaluation was the algebraic sum of these two losses. This is known as the Multibox loss and is defined in Equation (2).

(2) $$L_{conf}=\frac{1}{n_{positives}}(\sum_{positives}CE\;Loss+\sum_{hard\;negatives}CE\;Loss)$$

where

*L_conf_* = *confidence loss*
*n_positives_* = *total number of positives*
*CE Loss* = *Cross-Entropy loss*

### 2.3. Model Training

Validation datasets were used for monitoring the under-fitting and over-fitting of the detection model. The model training consisted of training the model using each object as a class. The three classes consisted of rock check dams, wattle check dams, and silt fence. The numbers of training and validation objects for rock check dams, wattle ditch checks, and silt fence were 3,820,138, and 1073, respectively, spread across 800 images. A total of 600 of the images were used for training and 200 were used for validation. Mean Average Precision (MAP) was used to report the accuracy of the validation set. The hyperparameters in Vinodababu’s framework were used in the model and re-tuning was not necessary since the existing hyperparameters were appropriate for the developed model [27]. The training was computed using the default parameters as described in Vinodababu’s framework [27] with a learning rate of 0.0001, a gradient clip of 0.5, and a batch size of 2.0. It was compiled on a Pytorch platform on an i7-8700 processor CPU and 32 GB RAM on NVIDIA GeForce GTX 1080Ti Platform under the Windows 10 Operating System.

### 2.4. Validation and Testing

**Figure 5:**
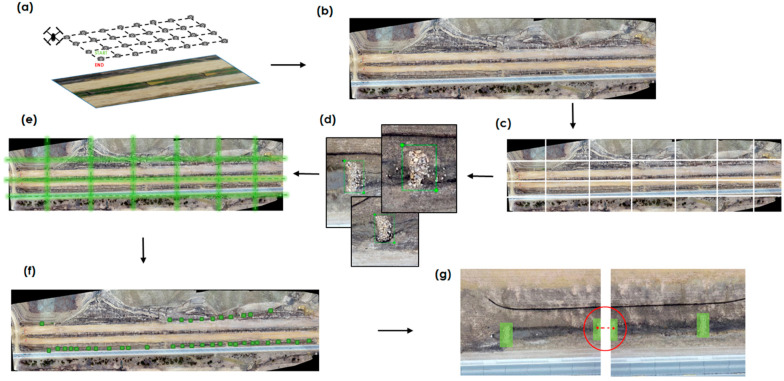
Detection flow chart: (**a**) aerial image capturing; (**b**) mosaic view layout preparation; (**c**) image tiling; (**d**) annotations; (**e**) image merging; (**f**) preliminary detection; (**g**) post-processing.

The results from the model produced bounding boxes around the objects that were smaller in shape and size when compared to the dimension of the original orthomosaic image. Hierarchical agglomerative clustering was carried out to merge detections into a single object as per their location on the original aerial image. This was computed and a threshold similar to Poddar et al.’s study submitted to the *Journal of Intelligent Transportation Systems*, which proposed to determine the total number of objects in the image [43]. The clustering was carried out with ‘city block’ affinity and a ‘single’ linkage mechanism and connectivity using the k_neighbors graph. Figure 5 illustrates the process flow chart of this study by showing each step of the methodology. The study focused on seven significant steps: (a) aerial image capturing with automated flights, (b) dataset preparation, (c) image tiling, (d) annotation, (e) image merging, (f) preliminary detection, and (g) post-processing.

## 3. Results and Discussion

Aerial inspections of temporary E&SC practices create an innovative approach in the field of construction stormwater management. These inspections can help contractors and owners meet environmental commitments and requirements by streamlining otherwise tedious and resource-intensive inspection practices. The results of this study showed that aerial inspections can capture high-quality images of implementations on-site, compared to photographs taken during traditional on-foot inspections. The comparison between aerial and ground acquired imagery provided a basis for understanding the limitations of the traditional inspections. The major limitation that was observed in traditional on-foot inspections was the narrow camera gate in-ground perspective images. This limitation causes difficulties in identifying the location of the practices and presents limited information on the deficiencies. Another observed limitation was the lack of geographic information on the ground perspective images, which disqualified them in forming a dataset for creating site plans and applying object detection principles.

**Figure 6:**
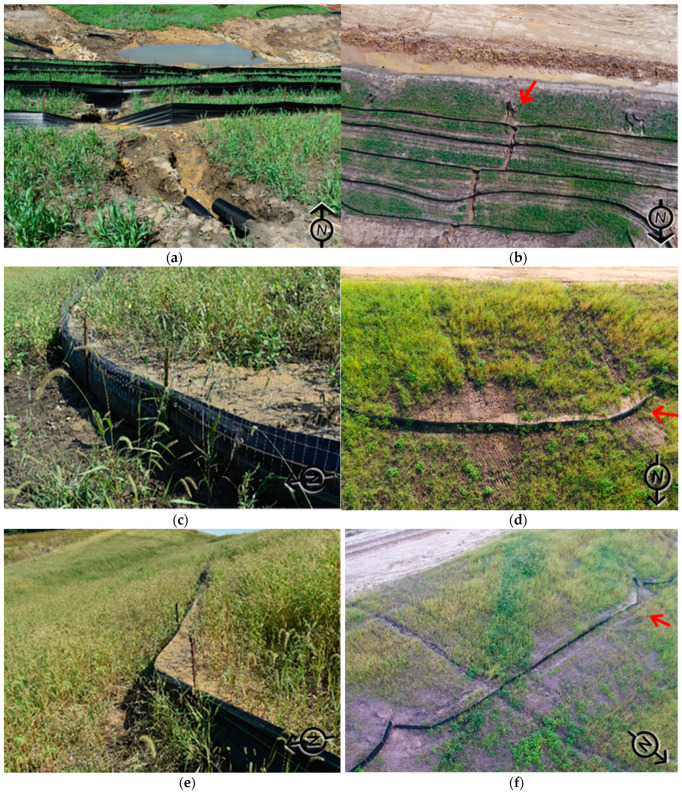
Comparison of silt fence inspection documentation: (**a**) overtopping failure (ground perspective); (**b**) overtopping failure (aerial perspective); (**c**) sediment accumulation (ground perspective); (**d**) sediment accumulation (aerial perspective); (**e**) sediment accumulation (ground perspective); (**f**) sediment accumulation (aerial perspective)

Figure 6 demonstrates a comparison of ground aerial images captured at the same sites. Figure 6a,c,e are ground images, and Figure 6b,d,f are aerial perspectives. The red arrows on the aerial photographs document the location and the perspective of the associated ground image. It can be observed from these figures above that the picture captured with the UAS not only provides a greater vantage of the deficiencies, but it also documents upstream issues that are causing the deficiencies. For example, Figure 6b clearly depicts how flow exiting through the slope drains is causing gully formation along the slope, resulting in the undermining of the installed silt fence sediment barrier. From this image, the inspector can see the magnitude of impact on the improperly installed slope drains and provide guidance to the contractor on extending the slope drains to the toe of the slope to prevent erosion. Figure 6d captures the source behind the sediment accumulation on the silt fence sediment barriers. The silt fence is receiving a significant amount of sediment accumulation due to gully erosion that is occurring on the slope. Figure 6e shows a gully on the slope that is resulting in sediment accumulation upstream of the silt fence. However, it is difficult to observe the entire failure along the channel with the on-foot camera perspective since the picture gate of the camera is limited. This comparison validated the idea of implementing aerial construction stormwater inspections for increasing efficiency by providing comprehensive data for record-keeping and site plan comparison. Aerial inspections provided an opportunity to track the progression of the construction site and individual practices by capturing weekly changes. Moreover, it was observed that aerial inspections save time and ensure the safety of inspection personnel since the UASs are controlled remotely by a remote pilot and a visual observer. The comparison of the two approaches showed that UASs can reach inspection areas rapidly and capture images from different perspectives with a wide picture gate. A comprehensive dataset for object detection can be easily formed with UAS-based imagery.

The study presented the capability of aerial inspections for improving current inspection procedures by developing automated inspection steps. High-resolution images with a wide-shot camera angle provided detailed information on the practices. Moreover, automated flights enabled user-friendly and rapid data collection, which was used for deep learning-based object detection in this study. Deep learning-based object detection brought a different and innovative perspective to aerial inspections by developing a model that can automatically detect the number, type, and location of temporary E&SC practices once aerial images are uploaded. With the use of this tool, inspectors and designers can easily keep track of site implementation by receiving continuous information from the site that can be compared with original plan sets. Future advances in object-based detection may lead to identifying failed practices or missing practices on a site. Furthermore, aerial imagery may be acquired using high-resolution satellite imagery, which completely removes the need for UAS based inspections.

**Figure 7:**
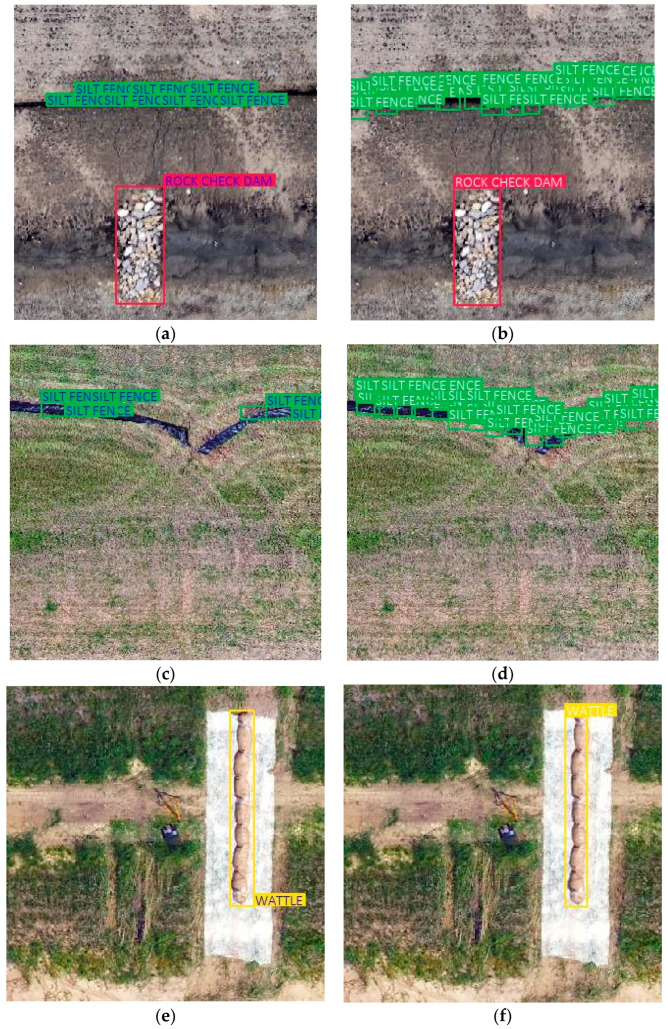
Comparison of validation results. (**a**) Original image; (**b**) validation result; (**c**) original image; (**d**) validation result; (**e**) original image; (**f**) validation result.

The results for this study were developed by using the default parameters as suggested by Vinodababu [27]. The necessary changes to the default parameters included using each object as a class, which were rock check dams, wattle ditch checks, and silt fece. The model was built from a data set of 800 images. As a result of this study, a perfect score on the MAP, which shows 100% accuracy on the results, was obtained as all the objects were detected by the algorithm with few false positive detections. Figure 7 shows the example validation results by comparing them with the original image. The white text in the images shows the validation results in Figure 7b,d,f and the colored text shows the original images that were labeled in the data preparation step in Figure 7a,c,e. The model was trained with original annotated images and validation results were produced by the model. It can be interpreted from this comparison that the model was successful at detecting practices. Table 1 quantifies the precision of the detection by presenting % MAP values for each practice.

However, the results for silt fence sediment barriers and ditch checks were showing the detection with a vast number of small boxes, as shown in Figure 7b,d. Hence, post-processing became necessary for detecting the practices as single objects.

**Figure 8:**
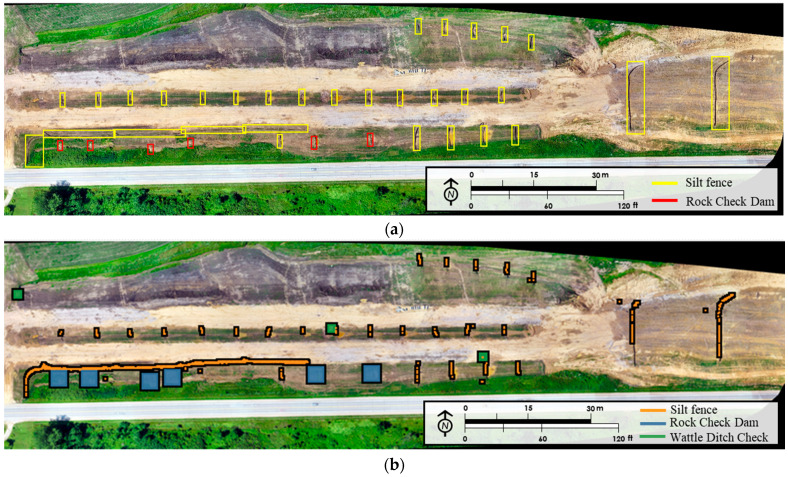
Post-processing results. (**a**) Training layout—18 July 2019; (**b**) result—18 July 2019.

In post-processing, the results were merged to display detection results in a single plan view and were compared with the original layouts. Figure 8 compares post-processing results for one of the layouts with the original layout. Figure 8a shows the training layout that was prepared on GIS for 18 July 2019 and compares it with the post-processing result after clustering detection results for each practice in Figure 8b. The comparison of the two layouts in Figure 8 showed that the training layout had 6 rock check dams, 0 wattle ditch checks, and 54 silt fence segments. The clustering results displayed 6 rock check dams, 5 wattle ditch checks, and 49 silt fence segments in the layout. This comparison shows that rock check dam detection was highly effective. However, the algorithm considered silt fence sediment barriers as a single silt fence, since the objects were very close to each other. The results also detected five wattle ditch checks which were not present in the original image in layout 18 July 2019. Two false wattle-ditch check detections occurred on corrugated HDPE pipe used for slope drain applications. Other false wattle detection results were due to algorithm-related issues.

**Figure 9:**
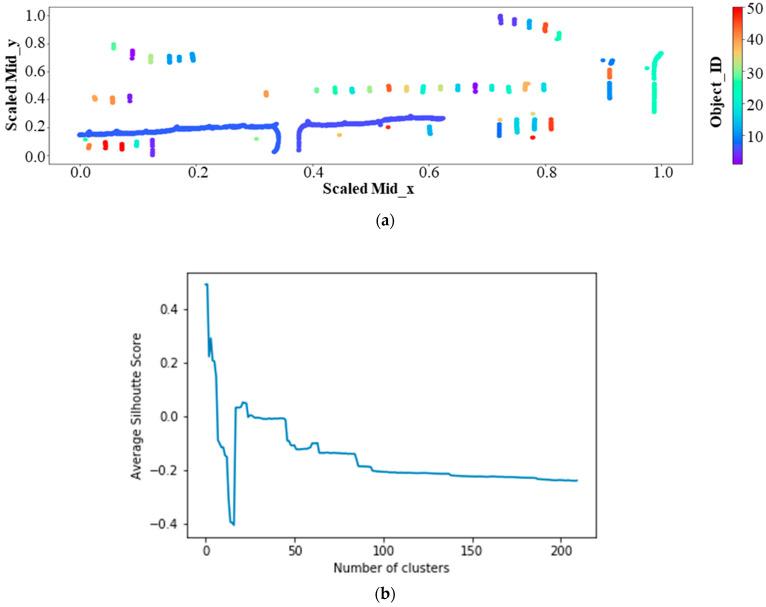
Post-processing result for silt fence segments. (**a**) Silt fence clustering result-18 July 2019; (**b**) silhouette score plot.

Figure 9a shows the silt fence post-processing results for the 18 July 2019 layout and Figure 9b shows the silhouette plot for the result represented in Figure 9a. Each unique silt fence segment was represented with a different color in Figure 9a. The average silhouette score shown in Figure 9b [44] was calculated by increasing the number of clusters from 2.0 onwards. Silhouette analysis was used to identify the separation distances between the clusters by scoring them in a range of [–1, 1]. The silhouette plot in Figure 9b displays the closeness of the cluster to the neighboring clusters. A lower average silhouette score means that the clusters are not well represented whereas a negative value of the same indicates that the members might be assigned to a wrong cluster. To determine the clusters, hierarchical clustering was adopted using ‘city block’ affinity among each member of a cluster, that is, with a ‘single’ linkage by adopting k-neighbors graph connectivity [44]. In Figure 9b, it can be observed that the average silhouette score shows a significant drop beyond 20 clusters. As per the domain knowledge of the locality and visual inspection, the largest drop in the average silhouette score beyond 20 clusters is taken as the total number of objects (silt fence sediment barriers or ditch checks) for the image.

## 4. Conclusions

UAS technology is increasingly being leveraged to provide rapid solutions across diverse disciplines due to its unique ability to capture visuals that are compatible with different applications such as photogrammetry, GIS, and deep learning. UAS is also expanding its usage in the construction industry by showing impressive progress for various purposes such as inspecting, monitoring, and surveying. This study compared aerial inspection images with on-foot inspection images. This comparison highlighted the opportunity to use UAS in inspections of E&SC practices with aerial perspective advantages, and efficiency in conducting inspections, all while meeting permit requirements. Aerial inspections have the capability to become an important data source for construction stormwater inspection. Remote pilots can stay at one location and capture significant amount of data from the site in an effective way. Although there are technical limitations such as low battery life and inoperable weather conditions, UASs can improve inspection implementation procedures and documentation with the use of appropriate equipment and technology. Future advances may lead to being able to use this technology as a fully autonomous tool.

The main goal of this study was to implement construction stormwater inspections by using UAS technology and providing various outputs for use of inspectors and designers with the integration of deep learning-based object detection. The results of the study emphasized the benefits of using deep learning-based object detection as a state-of-the-art technique for conducting efficient construction stormwater inspections.

In this study, deep learning-based object detection proved the capacity of aerial inspections to become an innovative approach by detecting temporary E&SC practices on a construction site. The SSD successfully identified rock check dams, wattle ditch checks, and silt fence on the images with complete accuracy. The post-processing step in this work enabled the display of detection results in the original layout, which provided the number and type of practices for plan set comparison with actual site applications. This study recommends further studies to focus on improve and optimize the post-processing tool. Furthermore, the determination of the length of the practices can be another contribution to this study to determine pay items and quantities. These pay items and quantities may include information on the length of geotextile fabric and the number of t-posts and other materials required to install silt fence sediment barriers and ditch checks. The model can be further refined by feeding it with additional data from different locations and providing a user-friendly interface for inspectors that detects practices with streamlined technical steps. Deficiencies to E&SC practices can be detected in future studies by training the code with more images that include typical deficiency types for each practice. This innovative approach will increase efficiency by saving time and resources required to conduct inspections while providing a greater amount of data for design and decision making. Moreover, it will widen the perspective of inspections and designs with its numerous useful features if it becomes applicable in the construction industry.

## Figures and Tables

**Table 1:** Mean average precision (MAP) values for detected practices.

	Detected Practices		
	Silt Fence	Rock Check Dam	Wattle Ditch Check
**% Mean Average** **Precision (MAP)**	100	100	100

## Data Availability

Some or all data, models, or code that support the findings of this study are available from the corresponding author upon reasonable request.
